# Dissecting Morphological and Functional Dynamics of Non‐Tumorigenic and Triple‐Negative Breast Cancer Cell Lines Using PCA and t‐SNE Analysis

**DOI:** 10.1002/cnr2.70257

**Published:** 2025-06-27

**Authors:** Yazan A. Almahdi, Eva R. Schwark, Aidan J. Mclaughlin, Besa Xhabija

**Affiliations:** ^1^ Department of Natural Science, College of Arts Sciences and Letters University of Michigan‐Dearborn Dearborn Michigan USA

**Keywords:** breast cancer, cell motility, digital holographic microscopy, MCF10A, MDA‐MB‐231, PCA, temporal dynamics, triple‐negative breast cancer, t‐SNE

## Abstract

**Background:**

Triple‐negative breast cancer (TNBC) poses significant challenges due to its aggressive nature and lack of targeted therapies. Understanding the cellular behaviors of TNBC is crucial for developing effective treatments.

**Aims:**

This study aims to compare the morphological characteristics of non‐tumorigenic MCF10A and aggressive MDA‐MB‐231 TNBC cell lines using advanced analytical techniques.

**Methods and Results:**

Advanced techniques such as Principal Component Analysis (PCA), t‐Distributed Stochastic Neighbor Embedding (t‐SNE), and digital holographic microscopy were utilized. Cellular features such as area, migration, motility, irregularity, and optical thickness were thoroughly analyzed over time. Our results revealed significant morphological differences between the MCF10A and MDA‐MB‐231 cell lines. Specifically, MDA‐MB‐231 cells displayed enhanced motility and a smaller, more variable size, attributes that may facilitate their invasive potential. In contrast, MCF10A cells exhibited larger sizes and more regular migration patterns, suggesting stability in structured tissue environments. Additionally, temporal analysis highlighted consistent phenotypic behaviors over time, with MDA‐MB‐231 cells demonstrating higher optical thickness and irregularity, indicating potential structural complexities associated with malignant transformation. Correlative analysis further confirmed these results by revealing connections between cell size, motility, and optical properties crucial for understanding cell behavior within their microenvironment.

**Conclusion:**

The profound differences in cellular dynamics between MCF10A and MDA‐MB‐231 cell lines underscore the unique adaptive mechanisms of TNBC cells. Our study provides valuable insights into the cellular foundations of TNBC aggressiveness, offering a foundation for future research aimed at understanding the mechanistic underpinnings of TNBC progression and therapeutic targeting.

## 1 Introduction

Breast cancer remains one of the most common malignancies among women worldwide, presenting significant variability in its biological behavior and clinical outcomes [1]. Understanding the cellular mechanisms underlying breast cancer phenotypes, particularly the aggressive triple‐negative breast cancer (TNBC), is crucial for developing targeted therapies and improving prognosis [2, 3, 4]. Morphological and functional analyses are critical for understanding cancer progression, as traits such as cell motility, migration patterns, and size directly influence the invasive and metastatic potential of cancer cells [5]. By investigating these characteristics, researchers can identify cellular behaviors that differentiate aggressive cancer subtypes like TNBC from normal tissue, providing insights into their ability to adapt to and invade diverse microenvironments. This study focuses on comparing the morphological and functional characteristics of non‐tumorigenic and tumorigenic breast cell lines, specifically the well‐characterized MCF10A and MDA‐MB‐231 lines, to elucidate the cellular behaviors that drive malignancy and metastatic potential in TNBC. Despite significant advances in breast cancer research, the specific morphological and functional mechanisms that distinguish TNBC cells from non‐tumorigenic counterparts, to the best of our knowledge, remain poorly understood. Addressing this gap is critical for identifying biomarkers and therapeutic targets that could mitigate TNBC's aggressive behavior. This study aims to fill this gap by leveraging advanced analytical techniques to uncover these distinctions in detail.

Advancements in cellular and molecular biology have provided insights into the distinct properties of cancer cells, including their ability to proliferate, migrate, and invade surrounding tissues [6, 7, 8]. The non‐tumorigenic MCF10A cell line, derived from human mammary epithelium, exhibits characteristics of normal epithelial cells and serves as an ideal model to contrast with cancerous phenotypes [9, 10, 11]. MCF10A was specifically chosen because it is a widely used, well‐characterized non‐tumorigenic breast epithelial cell line. Its phenotypic stability, suitability for long‐term studies, and extensive use in breast cancer research make it an established benchmark for comparing normal and cancerous cell behaviors [12]. On the other hand, the MDA‐MB‐231 cell line, a model for TNBC, is known for its high metastatic capacity and lack of estrogen, progesterone, and HER2 receptors, which typifies the TNBC profile associated with poor prognosis and limited treatment options [13].

Cell line models like MCF10A and MDA‐MB‐231 are extensively used in breast cancer research because they represent well‐characterized examples of non‐tumorigenic and triple‐negative breast cancer cells, respectively. MCF10A cells, derived from normal mammary epithelium, provide a benchmark for understanding baseline epithelial behavior, while MDA‐MB‐231 cells are an aggressive TNBC model widely studied for their metastatic potential [14]. These models are invaluable for investigating phenotypic differences that drive malignancy and metastatic progression in TNBC.

Employing techniques such as Principal Component Analysis (PCA) and t‐Distributed Stochastic Neighbor Embedding (t‐SNE), this study comprehensively analyzes the differences between these cell lines. PCA is a linear dimensionality reduction technique that simplifies complex datasets by identifying the main components contributing to variability [15]. In contrast, t‐SNE is a non‐linear method that captures local and global structures within data, enabling the visualization of clustering patterns [16]. These approaches provide an unbiased, high‐dimensional perspective on cellular behavior, overcoming the limitations of traditional analyses that evaluate only one or two features at a time (e.g., plotting cell area versus migration distance in a 2D scatter plot). Utilization of Digital Holographic Microscopy (DHM), a label‐free imaging technique, further enhances this approach by allowing real‐time, non‐invasive quantification of cellular morphology and dynamics, helping in preserving native cell behavior while at the same time generating richer datasets [17]. Altogether, these advanced computational and imaging methods enable a deeper, more nuanced understanding of TNBC aggressiveness, revealing phenotypic heterogeneity that might be overlooked with conventional techniques. These methods are particularly suited to exploring the phenotypic and functional differences between non‐tumorigenic MCF10A and aggressive MDA‐MB‐231 cells, providing insights into the adaptive mechanisms of triple‐negative breast cancer. These analytical methods enable the visualization and quantification of high‐dimensional data to uncover underlying patterns and associations that may not be apparent with traditional bi‐dimensional analysis. Through a series of detailed morphological assessments, we aim to explore how cellular size, shape, and motility contribute to the aggressive behavior exhibited by TNBC cells. Conventional assays (e.g., flow cytometry, fluorescence microscopy) typically track one or two features at a time and often rely on labels that can perturb cell behavior. By combining label‐free digital holographic microscopy with PCA and t‐SNE, we instead capture—and unbiasedly visualize—high‐dimensional, dynamic cellular phenotypes.

The integration of temporal dynamics and correlative analyses further enhances our understanding of how these cells adapt over time and interact with their microenvironment, thereby providing valuable insights into the intrinsic and extrinsic factors that govern cancer cell behavior. This study sheds light on the fundamental differences between non‐tumorigenic and TNBC cells and highlights potential biomarkers and therapeutic targets. As breast cancer research continues to advance towards personalized medicine, it is imperative to dissect the cellular and molecular distinctions between different breast cancer subtypes. This manuscript presents novel findings that contribute to the broader understanding of TNBC's aggressive nature and lays the groundwork for future studies to mitigate its impact on patient health. Through rigorous comparative analysis and innovative visualization techniques, we offer a detailed portrayal of the phenotypic and functional disparities that define non‐tumorigenic and triple‐negative breast cancer cells, paving the way for advancements in clinical strategies and therapeutic interventions.

## 2 Materials and Methods

### 2.1 Cell Culture and Digital Holographic Microscopy Analysis

Digital Holographic Microscopy (DHM) is a label‐free, quantitative imaging technique that captures real‐time cellular morphology and dynamics by recording interference patterns of coherent light passing through or reflected from cells. This approach enables the non‐invasive study of live cells, preserving their natural behavior while providing high‐resolution quantitative phase images [18, 19, 20, 21]. Unlike fluorescence microscopy, which often requires labeling that can perturb cellular behavior, DHM allows for longitudinal observations under near‐physiological conditions. These characteristics make DHM particularly well‐suited for studies requiring the tracking of cellular motility, migration, and structural changes over time. DHM operates by capturing interference patterns generated as coherent light passes through cells, which are then computationally reconstructed to provide both phase and amplitude information [22]. The resulting quantitative phase images enable measurement of cellular morphology and dynamic behaviors with high spatial and temporal resolution. Holominitor uses Fourier‐based digital holography [22, 23] to extract key features, following established methodologies for refractometry and quantitative analysis of adherent cells [24].

In this study, DHM was employed to investigate the morphological and functional differences between non‐tumorigenic MCF10A and aggressive triple‐negative breast cancer MDA‐MB‐231 cell lines. The ability of DHM to provide label‐free, real‐time imaging allowed for a comprehensive analysis of key parameters such as cell size, motility, irregularity, and optical thickness without disrupting cellular integrity. These unique features of DHM facilitated the temporal tracking of cellular behaviors and provided robust datasets for subsequent high‐dimensional analysis using PCA and t‐SNE. MCF10A (ATCC CRL‐10317) and MDA‐MB‐231 (ATCC HTB‐26) were seeded at approximately 50% confluency in MEBM Basal Medium supplemented with BPE, hEGF, Insulin, hydrocortisone, GA‐1000 (Lonza, Basel, Switzerland) and RPMI1640, 10% FBS, 5% Penicillin/Streptomycin, Insulin (1 mg/mL, Gibco, New York, USA) at 37°C with 5% CO_2_. The study employed a HoloMonitor M4, manufactured by Phase Holographic Imaging (PHI, Lund, Sweden). The Hstudio software package from PHI enabled time‐lapse phase imaging, image manipulation, segmentation, and data examination. Images were captured at 15‐min intervals, starting 24 h after cell seeding. Temporal changes in cellular morphology, including features such as area, motility, and irregularity, were analyzed using time‐lapse data generated by Digital Holographic Microscopy (DHM). Quantitative measurements were extracted using HStudio software and subsequently processed in Python for statistical analysis. Temporal patterns were assessed through feature‐specific scatter plots, and independent two‐sample t‐tests were applied to evaluate significant differences over time between the cell lines. PCA and t‐SNE were used to visualize temporal clustering and identify trends in high‐dimensional data. The images from each well were divided into distinct sections, identified, and the number of cells was determined using the Cell Count function of the HStudio software. For the tracking as mentioned above assays, cells were cultured following standard conditions and seeded in 24‐well plates (Sarstedt, Hildesheim, Germany).

### 2.2 Data Collection and Processing

The current study thoroughly compared MCF10A and MDA‐MB‐231 cell lines, which are representative of healthy mammary epithelial cells and aggressive triple‐negative breast cancer cells, respectively. Our approach involved several steps. The HoloMonitor M4 software collected multiple cellular features, including area, irregularity, migration, motility, migration directness, and optical thickness. These specific features were selected for analysis based on their biological relevance to cell morphology and motility, their statistical variability between the two cell lines, and to avoid redundancy. Features such as centroid positions were excluded as they did not provide direct biological insights into the study objectives. This selection strategy ensured a focused and interpretable analysis of phenotypic differences between MCF10A and MDA‐MB‐231 cells. Then, data import and cleaning began with the export of raw data generated from Digital Holographic Microscopy (DHM), which were then imported into Python using the pandas.read_excel() function. Cleaning included standardizing data formats, such as converting the ‘time’ column (initially in string format) into a date time object using pandas.to_datetime() and further converting time values into minutes from the start of the day for ease of temporal analysis. Any missing or inconsistent data points were identified and appropriately handled to ensure data integrity. Dimensionality reduction was performed using Principal Component Analysis (PCA) and t‐Distributed Stochastic Neighbor Embedding (t‐SNE). PCA simplified the high‐dimensional dataset by identifying features that contributed the most variance between cell lines, enabling the reduction of data into principal components for more accessible visualization and analysis. t‐SNE complemented PCA by mapping the data into a lower‐dimensional space, which helped identify clustering patterns and subtle differences between the cell lines. Data visualization included the use of scatter plots, box plots, and heatmaps to present trends and differences in cellular features such as size, motility, and optical thickness. Temporal dynamics were visualized by plotting feature values over time to observe consistent behavioral patterns across experimental conditions. These visualizations were generated using Python libraries such as matplotliband seaborn. Finally, statistical analysis was conducted to quantitatively compare the two cell lines. Independent two‐sample t‐tests were applied using scipy.stats.ttest_ind() to evaluate significant differences in cellular features between MCF10A and MDA‐MB‐231 cells. Results from these statistical analyses were systematically organized and exported for further interpretation. This workflow ensured robust and comprehensive analysis of the morphological and functional differences between non‐tumorigenic and TNBC cell lines. Moreover, to ensure comparability of measurements across different feature scales, raw values were normalized using a min‐max scaling approach, transforming feature values to a range of [0,1] to prevent biases due to differing numerical ranges. Although all input features were scaled to [0,1], the resulting principal component scores may lie outside this interval, reflecting their status as variance‐maximizing linear combinations. Additionally, to enhance data robustness and reduce the influence of extreme outliers, we applied an outlier removal step using the interquartile range (IQR) method. Any data points falling beyond 1.5 times the IQR from the first and third quartiles were excluded from subsequent analyses. These preprocessing steps ensured that all downstream statistical comparisons and dimensionality reduction techniques were performed on a standardized dataset. To ensure consistency and comparability across different cellular features, all quantitative values were normalized to a common scale using Min‐Max Normalization. This approach standardizes the data by adjusting values to a fixed range, preserving relative differences while allowing meaningful comparisons between MCF10A and MDA‐MB‐231 cells. Normalized features include Avg. Area, Avg. Migration, Avg. Motility, Avg. Irregularity, Avg. Migration Directness, and Avg. Optical Thickness.

For the correlation analysis in Figure 4, all values from both MDA‐MB‐231 and MCF10A cells were combined to compute the correlations. This approach allows for an overall assessment of how morphological features relate across both cell types, identifying general trends in cellular behavior. The Pearson correlation coefficient was used to assess linear relationships between features.

### 2.3 PCA and t‐SNE Plots

We first applied Principal Component Analysis (PCA) and t‐Distributed Stochastic Neighbor Embedding (t‐SNE) to visualize the high‐dimensional dataset in a two‐dimensional space. PCA reduces the dimensionality of the dataset while retaining features that explain the most variance between cell lines, simplifying the data for clearer interpretation. This approach is particularly helpful for identifying key features that distinguish the MCF10A and MDA‐MB‐231 cell lines. t‐SNE complements PCA by preserving both local and global structures in the data and enabling the visualization of clustering patterns that reflect subtle phenotypic differences between the cell lines. The two techniques together provide a robust framework for analyzing and illustrating the unique morphological and functional behaviors of 1206 MCF10A and 1542 MDA‐MB‐231 cells. The results of these procedures were plotted using matplotlib.pyplot.scatter() to create scatter plots with cells color‐coded by cell line.

### 2.4 Box Plots

To visualize the distribution of individual features, box plots were generated using seaborn. boxplot(). Nine features were selected for this analysis, including “Avg. Area (μm^2^)”, “Avg. Migration (μm)”, “Avg. Motility (μm)”, “Avg. Irregularity”, “Avg. Migration directness (migration vs motility)”, “Avg. Optical thickness avg (μm)”, “No. tracked cells”, “Avg. Motility speed (μm/h)”, and “Avg. Optical volume (μm^3^)”. Moreover, scatter plots of feature values over time were generated to examine temporal trends using matplotlib.pyplot.scatter(). Each data point represented a single time point, and the time axis was represented in minutes from the start of the day.

### 2.5 Heat Maps, Scatter Plot Matrix and Multimodal Visualization

Correlations between different features were visualized using heatmaps generated with the Python library seaborn. We utilized a Pearson correlation matrix to compute pairwise correlations between cellular features. A diverging color palette was applied to the heatmap to represent the strength and direction of the correlations, with positive correlations shown in one color and negative correlations in another. Additionally, a scatter plot matrix was constructed using the seaborn.pairplot() function to examine pairwise relationships between cellular features such as ‘No. tracked cells,’ ‘Avg. Area (μm^2^),’ ‘Avg. Migration (μm),’ ‘Avg. Motility (μm),’ and ‘Avg. Optical volume (μm^3^).’ Each scatter plot represented the relationship between two features, while the diagonal plots displayed the distribution of individual features. Then, in order to identify the most variable morphological and motility‐related features distinguishing MCF10A and MDA‐MB‐231 cell lines, we generated the variance of each feature across all samples. This was calculated using the var.() function in R and log‐transformed in order to enhance feature differentiation. The feature with the lowest variance was excluded from further analysis, however the remaining features were ranked from highest to lowest variance to prioritize those with the greatest discriminatory potential.

In order to characterize the phenotypic differences between MCF10A and MDA‐MB‐231 cell lines, we employed a multi‐faceted analytical approach. Initially, we ranked cellular features by their absolute Cohen's d effect sizes, revealing migration directness, area, and irregularity as significantly elevated in MCF10A cells, while optical thickness, volume, and motility predominated in MDA‐MB‐231 cells (*p* < 0.001). To better visualize these contrasts, we constructed a forest plot displaying effect directions with confidence intervals, categorizing differences as very large (triangles, |*d*| ≥ 1.2), large (diamonds), medium (arrows), or small (circles). The circular profile arrangement of these features offered a complementary perspective, with bar heights representing effect magnitudes and colors indicating the dominant cell line. For deeper insights into the standardized relationships, we generated a heatmap using *Z*‐scores, which highlighted the stark contrast between the phenotypes. The radar chart further emphasized this dichotomy by plotting effect sizes in a polar coordinate system, creating distinctive “fingerprints” for each cell line. Finally, we examined the underlying distributions through ridgeline plots, which revealed not just differences in central tendency but also in variability and skewness—particularly notable in optical thickness distributions where MDA‐MB‐231 cells showed greater heterogeneity. All analyses and visualizations were performed using R (version 4.2.0) with the ggplot2, gridExtra, and ggridges packages.

### 2.6 Statistical Analysis

For statistical analysis, independent two‐sample t‐tests were performed for each numerical feature using scipy.stats.ttest_ind(). This function calculates the *t*‐test for the means of two independent samples and returns the T statistic and the two‐tailed *p*‐value. The results of the *t*‐tests were organized in a DataFrame, which was exported as an Excel file using pandas.DataFrame.to_excel(). Data processing, statistical computations, and visualizations were conducted in Python, leveraging various data analysis and visualization libraries. This approach allowed us to elucidate significant differences between the MCF10A and MDA‐MB‐231 cell lines, thereby contributing valuable insights into the aggressive nature of the MDA‐MB‐231 cell line.

## 3 Results

### 3.1 The Analysis Using Multidimensional Scaling Revealed Characteristics of Tumorigenic and Triple Negative Breast Cancer Cell Lines

**FIGURE 1:**
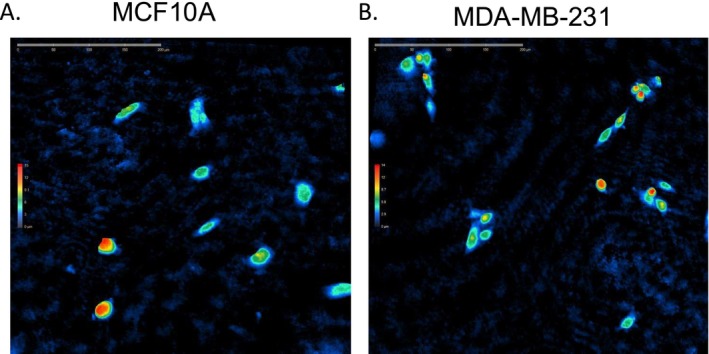
Digital Holographic Microscopy (DHM) images of MCF10A and MDA‐MB‐231 cell lines. The 3D topographical representation of cellular morphology is shown, with color‐coded height maps indicating optical thickness (in micrometers). Warmer colors (red and yellow) correspond to areas of higher optical thickness, while cooler colors (blue) indicate lower optical thickness. The left panel represents the non‐tumorigenic MCF10A cells, while the right panel shows the triple‐negative breast cancer MDA‐MB‐231 cells. The scale bars represent 100 μm. These images highlight the morphological and structural differences between the cell lines. These are raw phase images acquired via label‐free digital holographic microscopy (DHM); cell boundaries appear subtle due to the nature of this imaging modality.

**FIGURE 2:**
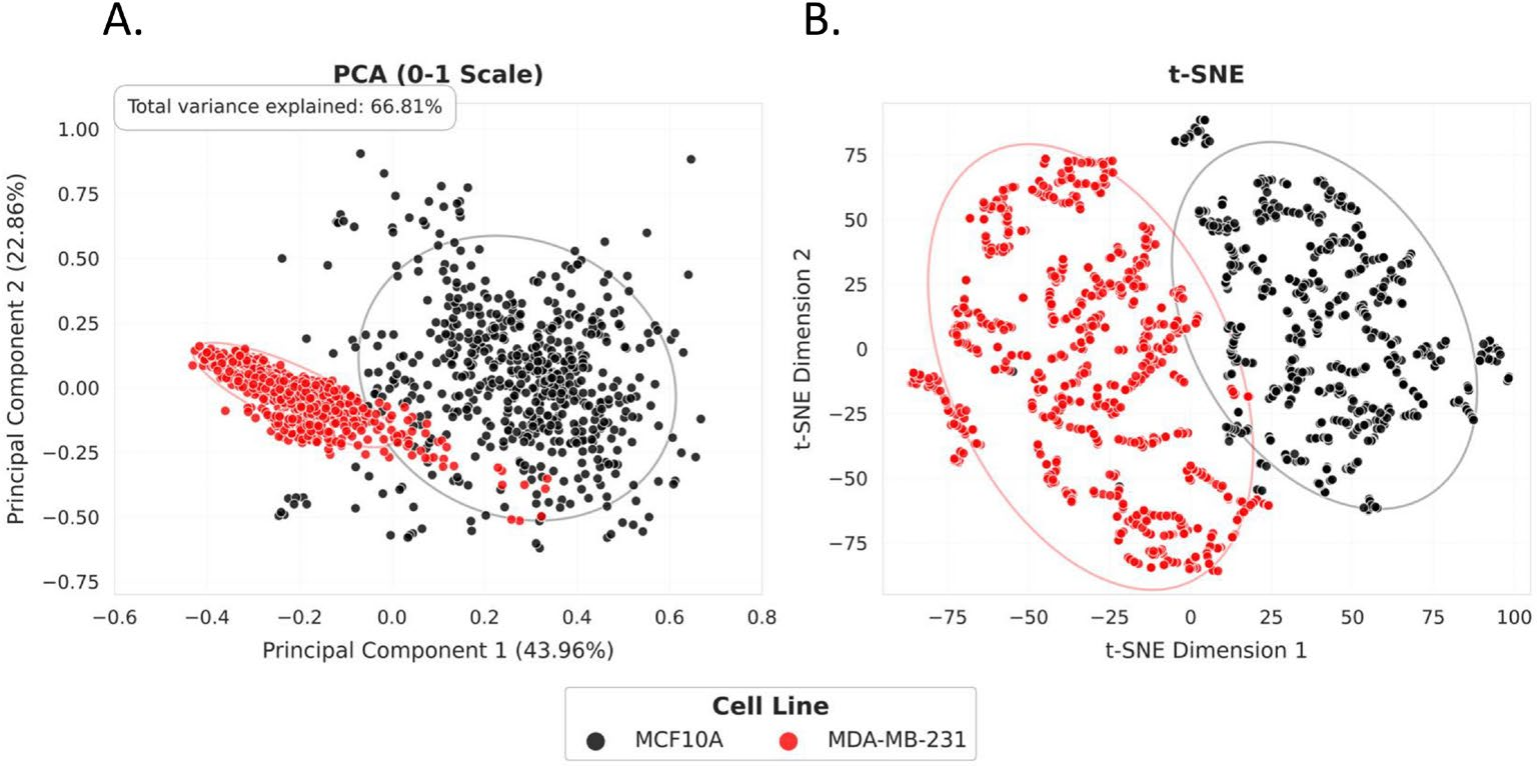
PCA and t‐SNE plots of MCF10A and MDA‐MB‐231 cells. Each point represents a cell, with black points representing MCF10A cells and red points representing MDA‐MB‐231 cells. (A) In the PCA plot the *x* and *y* axes represent the first two principal components. (B) The t‐SNE plot of MCF10A and MDA‐MB‐231 cells. Each point represents a cell, with black points representing MCF10A cells and red points representing MDA‐MB‐231 cells. The *x* and *y* axes represent the two t‐SNE dimensions.

Figure 1 shows representative digital holographic microscopy images of the non‐tumorigenic MCF10A (left) and triple‐negative MDA‐MB‐231 (right) cell lines. To improve visualization of individual cell boundaries, we have included Figure S1, which overlays computationally segmented cell outlines onto the original DHM phase images for both cell lines. This complements the unmodified phase maps shown in Figure 1 and enhances interpretability while preserving the label‐free imaging approach. We employed Principal Component Analysis (PCA) and t‐Distributed Stochastic Neighbor Embedding (t‐SNE) to explore the differences between the non‐tumorigenic MCF10A and the aggressive breast cancer MDA‐MB‐231 cell lines, Figure 2. In Figure 2A, the PCA plot illustrates some degree of separation between the MCF10A and MDA‐MB‐231 cell lines based on the first two principal components (PCA1 and PCA2). This separation indicates distinct underlying patterns in the dataset, with the MDA‐MB‐231 cell line displaying generally higher values along both PCA1 and PCA2. Such positioning suggests that this cell line may possess unique molecular or phenotypic traits distinguishing it from the MCF10A cell line. Despite the discernible separation, the overlap between the data points of the two cell lines suggests the presence of shared characteristics or less pronounced differences in some features captured by PCA. The plot reveals significant sources of variation within the dataset but also highlights the complexity and subtleties of the differences between these cell lines.

The PCA and t‐SNE plots revealed distinct clustering patterns that highlight differences between the MCF10A and MDA‐MB‐231 cell lines, 2. PCA provided an overview of the primary sources of variance within the dataset, allowing us to identify the most distinguishing features of the two cell lines. On the other hand, the t‐SNE plot provided a more defined visual distinction by preserving both local and global data relationships, making it particularly useful for revealing subtle phenotypic differences that align with the aggressive behavior of MDA‐MB‐231 cells. The use of PCA and t‐SNE enables a more refined exploration of high‐dimensional cellular feature data compared to conventional methods. Traditional analyses such as fluorescence microscopy, scratch wound assays, and migration tracking often rely on pre‐selected parameters or endpoint measurements that may not fully capture phenotypic diversity. In contrast, PCA provides an unbiased approach to identifying the primary drivers of variation, while t‐SNE preserves complex relationships within the data, highlighting subtle clustering patterns that might be overlooked in standard statistical analyses. These results demonstrate the power of integrating dimensionality reduction techniques with real‐time, label‐free imaging to gain deeper insights into the heterogeneity of TNBC cell behavior. In this plot, the MDA‐MB‐231 cell line forms a well‐defined, separate cluster from the MCF10A cell line, emphasizing the distinct nature of the cancerous cells. This distinct clustering suggests robust differences in the data features between the two cell lines, underscoring specific cellular behaviors or properties unique to the MDA‐MB‐231 cell line.

### 3.2 Comparative Analysis of Cell Dynamics Between Non‐Tumorigenic and Triple‐Negative Breast Cancer Cell Lines

**FIGURE 3:**
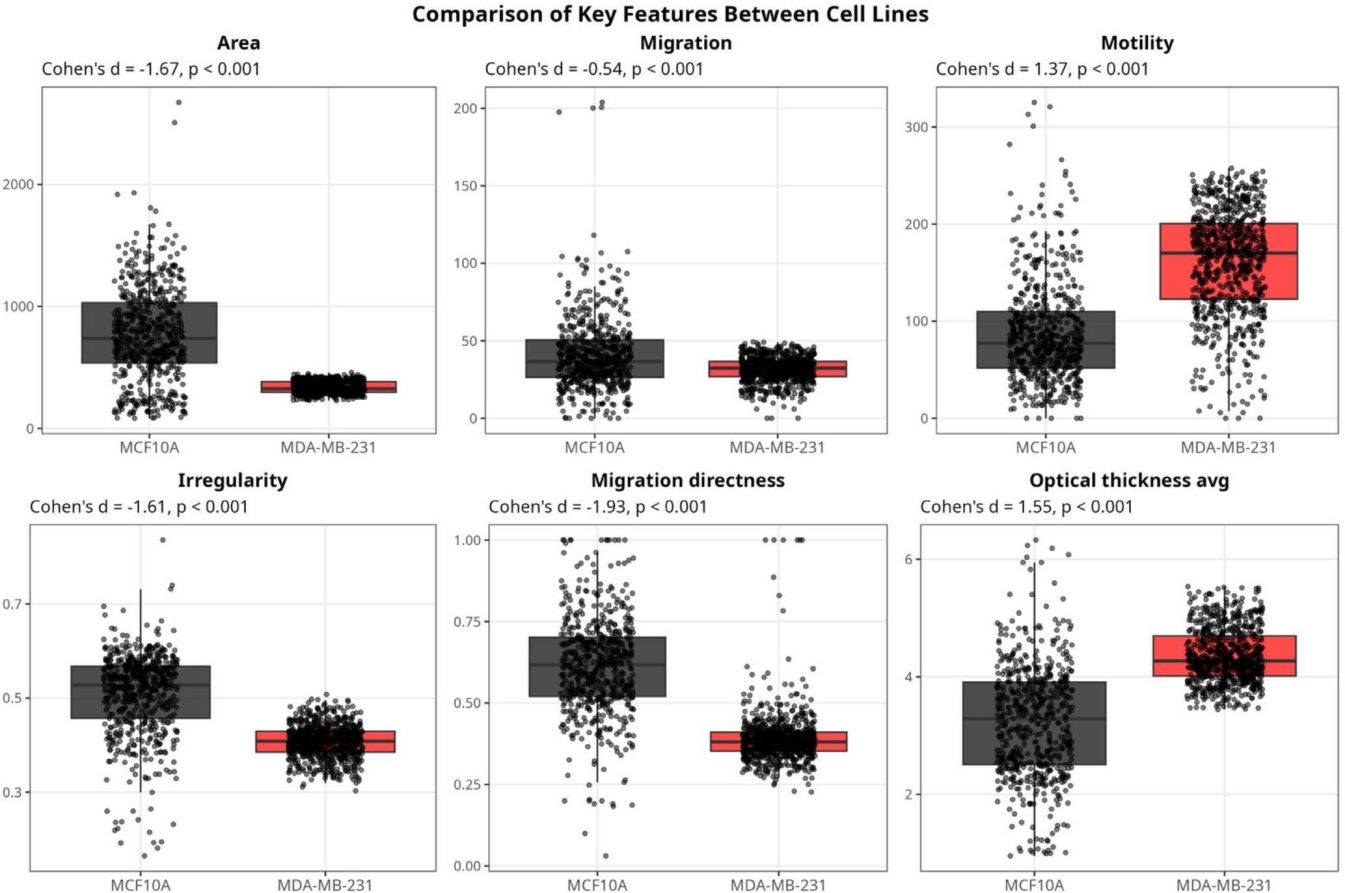
Box plots for six selected features (Area, Migration, Motility, Irregularity, Migration directness, and Optical thickness avg). Each box plot presents the distribution of a feature for the two cell lines, with black representing MCF10A and red representing MDA‐MB‐231. Statistical significance is indicated by *p*‐values, and effect sizes are shown as Cohen's *d* values.

The current study utilized a range of morphological and functional parameters to compare the non‐tumorigenic breast epithelial cell line MCF10A with the metastatic breast cancer cell line MDA‐MB‐231, as depicted in Figure 3. Detailed quantitative analyses of average cellular area, migration, motility, irregularity, migration directness, and optical thickness revealed distinct physiological and behavioral patterns between the two cell lines. Our analysis shows that MCF10A cells have a significantly larger average cellular area (765.47 μm^2^) than MDA‐MB‐231 cells (335.40 μm^2^), Figure 3. Despite their non‐tumorigenic nature, MCF10A cells exhibit a larger physical footprint, which contrasts with the smaller, yet more variable size of the aggressive MDA‐MB‐231 cells. This size variability in MDA‐MB‐231 cells may facilitate their migration through diverse microenvironments, enhancing their invasive potential. Interestingly, MCF10A cells demonstrated a slightly greater average migration distance (40.87 μm) than MDA‐MB‐231 cells (31.55 μm). In contrast, MDA‐MB‐231 cells exhibited higher motility (161.18 μm) compared to MCF10A cells (87.74 μm), underlining the former's enhanced capability for sustained movement, crucial for metastatic dissemination, Figure 3. The enhanced motility observed in MDA‐MB‐231 cells, combined with their smaller and more variable size, highlights traits that facilitate invasive behavior [25]. These features serve as potential biomarkers for distinguishing aggressive TNBC cells from non‐tumorigenic cells. Moreover, the motility mechanisms underlying these traits represent promising therapeutic targets aimed at reducing cellular invasiveness.

Structural irregularity measurements indicated that MCF10A cells (0.509) are more irregular than MDA‐MB‐231 cells (0.407). This higher irregularity in MCF10A could be reflective of a heterogeneous cell population or different cell cycle stages captured during analysis. Moreover, MCF10A cells displayed a higher migration directness ratio (0.615) compared to MDA‐MB‐231 cells (0.390) (Figure 3), suggesting that MCF10A cells migrate more linearly towards targets, potentially advantageous in structured tissue contexts. In contrast, MDA‐MB‐231's lower directness ratio implies a more random and exploratory migration pattern, indicative of their metastatic nature. The optical thickness was slightly greater in MDA‐MB‐231 cells (4.36 μm) than in MCF10A cells (3.24 μm). This increased thickness in MDA‐MB‐231 cells might suggest denser or more complex intracellular structures, which are characteristic features associated with malignant transformations and the aggressive behavior of cancer cells.

### 3.3 Statistical Analysis of Morphological and Functional Differences

In order to assess the morphological and functional differences between two cell lines, independent *t*‐tests and Cohen's *d* effect size calculations were conducted. The results as shown in Table 1 revealed highly significant differences (*p* < 10^−18^) for all six measured metrics, including Avg_Area, Avg_Migration, Avg_Motility, Avg_Irregularity, Avg_Directness, and Avg_Optical_Thickness. Cohen's *d* values indicated large effect sizes, underscoring substantial differences between the two cell lines. These results quantitatively confirm that smaller cell size and higher motility, combined with irregular migration patterns, are hallmarks of the invasive phenotype exhibited by MDA‐MB‐231 cells. These quantitative findings complement the observed trends in motility and size metrics reported below. The significant differences observed between MCF10A and MDA‐MB‐231 were confirmed through statistical analysis (Table 1), with *p*‐values below 10^−18^ across key metrics such as area, migration, motility, and optical thickness. Additionally, Cohen's d effect sizes indicate that these differences are biologically meaningful, further validating the robustness of the observed phenotypic variations between the two cell lines (Table 2).

**TABLE 1:** The statistical analysis of morphological and behavioral metrics in MCF10A and MDA‐MB‐231 cell lines.

Feature	MCF10A mean	MCF10A SD	MDA‐MB‐231 mean	MDA‐MB‐231 SD	Cohen's *d*	*p*‐value	Significance
Migration directness	0.61	0.15	0.39	0.08	−1.93	< 0.001	***
Area	765.47	385.47	335.4	50.63	−1.67	< 0.001	***
Irregularity	0.51	0.09	0.41	0.04	−1.61	< 0.001	***
Optical thickness	3.24	0.96	4.36	0.46	1.55	< 0.001	***
Motility	87.74	52.41	161.18	54.2	1.37	< 0.001	***
Migration	40.87	24.18	31.55	8.09	−0.54	< 0.001	***

**TABLE 2:** Phenotypic features ranked by magnitude of difference between MCF10A and MDA‐MB‐231 breast cell lines.

Rank	Feature	MCF10A mean	MCF10A SD	MDA‐MB‐231 mean	MDA‐MB‐231 SD	% Difference	Cohen's d	*p*‐value	Significance	Higher In
1	Migration directness	0.61	0.15	0.39	0.08	−44.69	−1.93	< 0.001	***	MCF10A
2	Area	765.47	385.47	335.4	50.63	−78.13	−1.67	< 0.001	***	MCF10A
3	Irregularity	0.51	0.09	0.41	0.04	−22.24	−1.61	< 0.001	***	MCF10A
4	Optical thickness avg	3.24	0.96	4.36	0.46	29.49	1.55	< 0.001	***	MDA‐MB‐231
5	Motility	87.74	52.41	161.18	54.2	59.01	1.37	< 0.001	***	MDA‐MB‐231
6	Optical volume	2826.35	1863.17	1414.8	165.26	−66.56	−1.14	< 0.001	***	MCF10A
7	Motility speed	37.88	14.15	26.77	3.64	−34.36	−1.14	< 0.001	***	MCF10A
8	Centroid pos Y	342.92	109.83	287.69	45.78	−17.51	−0.69	< 0.001	***	MCF10A
9	Centroid pos X	234.62	125.85	285.93	43.92	19.71	0.57	< 0.001	***	MDA‐MB‐231
10	Migration	40.87	24.18	31.55	8.09	−25.73	−0.54	< 0.001	***	MCF10A

### 3.4 Temporal Dynamics of Cell Behavior in Non‐Tumorigenic and Triple‐Negative Breast Cancer Cell Lines

**FIGURE 4:**
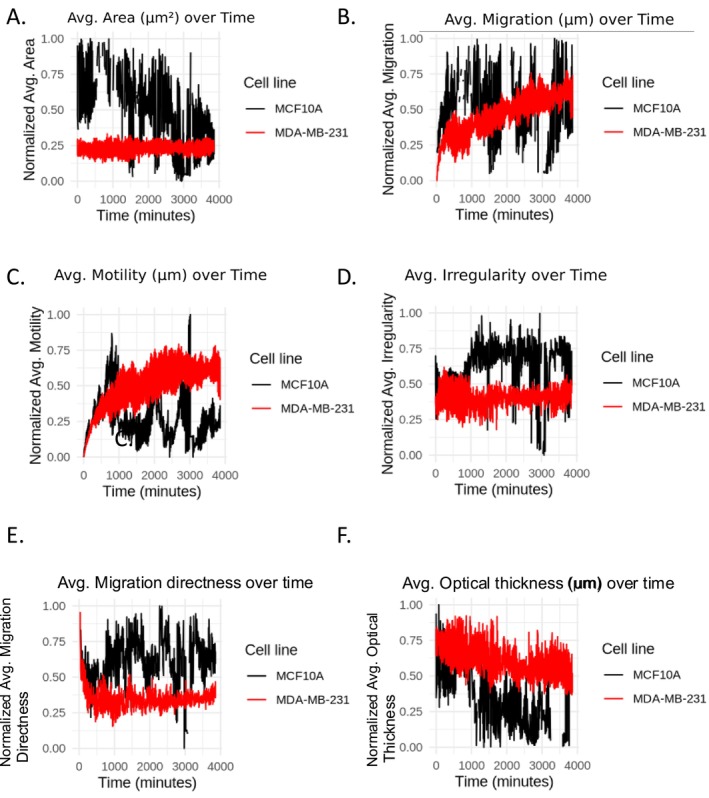
Line plots over time for six selected features. Each plot presents the temporal dynamics of a feature for the two cell lines, with black representing MCF10A and red representing MDA‐MB‐231. The *x*‐axis represents time in minutes, and the *y*‐axis represents normalized feature values. (A) Avg. Area (μm^2^), (B) Avg. Migration (μm), (C) Avg. Motility (μm), (D) Avg. Irregularity, (E) Avg. Migration directness, and (F) Avg. Optical thickness avg. (μm) over time.

A series of normalized plots comparing various cellular features of the MDA‐MB‐231 and MCF10A cell lines over a defined time series are shown in Figure 4. Each subplot in Figure 4 (labeled A to F) illustrates distinct aspects of cellular behavior, providing insights into the dynamic changes and differences between the non‐tumorigenic and cancerous cell lines. The data reveals that MCF10A cells exhibit a consistently larger average area than MDA‐MB‐231 cells, which remain smaller and more uniform in size (Figure 4A). This size difference suggests that MCF10A maintains a more structured morphology, whereas MDA‐MB‐231 may undergo shape adaptations relevant to cancer cell invasion. Additionally, MCF10A cells also display greater migration distances compared to MDA‐MB‐231 (Figure 4B), indicating that they tend to travel further over time.

In contrast, while MDA‐MB‐231 cells exhibit less migration distance overall, they demonstrate higher motility, meaning they move more frequently (Figure 4C). Interestingly, MCF10A cells seem to exhibit greater irregularity over time, fluctuating in shape, while MDA‐MB‐231 maintains a more stable morphology (Figure 4D). This variability in MCF10A could be attributed to differences in cellular structure and function. Similarly, MCF10A cells exhibit higher migration directness (Figure 4E), meaning they move more linearly, while MDA‐MB‐231 cells migrate in a more random, exploratory manner.

Finally, MDA‐MB‐231 cells seem to have a consistently higher optical thickness compared to MCF10A (Figure 4F), suggesting increased intracellular density or compositional differences that may contribute to their aggressive phenotype. These normalized plots collectively highlight key phenotypic differences in MDA‐MB‐231 and MCF10A cells, with implications for understanding cancer cell behavior. Within‐cell variability in optical thickness was quantified and is summarized in Table S1 (mean, SD, and CV for each cell line), revealing a higher coefficient of variation CV in MCF10A cells (0.295) than in MDA‐MB‐231 cells (0.106). The stable expression of features such as motility, migration directness, and optical thickness in MDA‐MB‐231 cells underscores their phenotypic adaptations that may facilitate tumor progression. These traits, particularly their persistent motility and structural stability, could be further investigated as potential biomarkers and targeted to mitigate TNBC aggressiveness.

### 3.5 Correlative Analysis of Morphological and Functional Parameters in Breast Cell Lines

**FIGURE 5:**
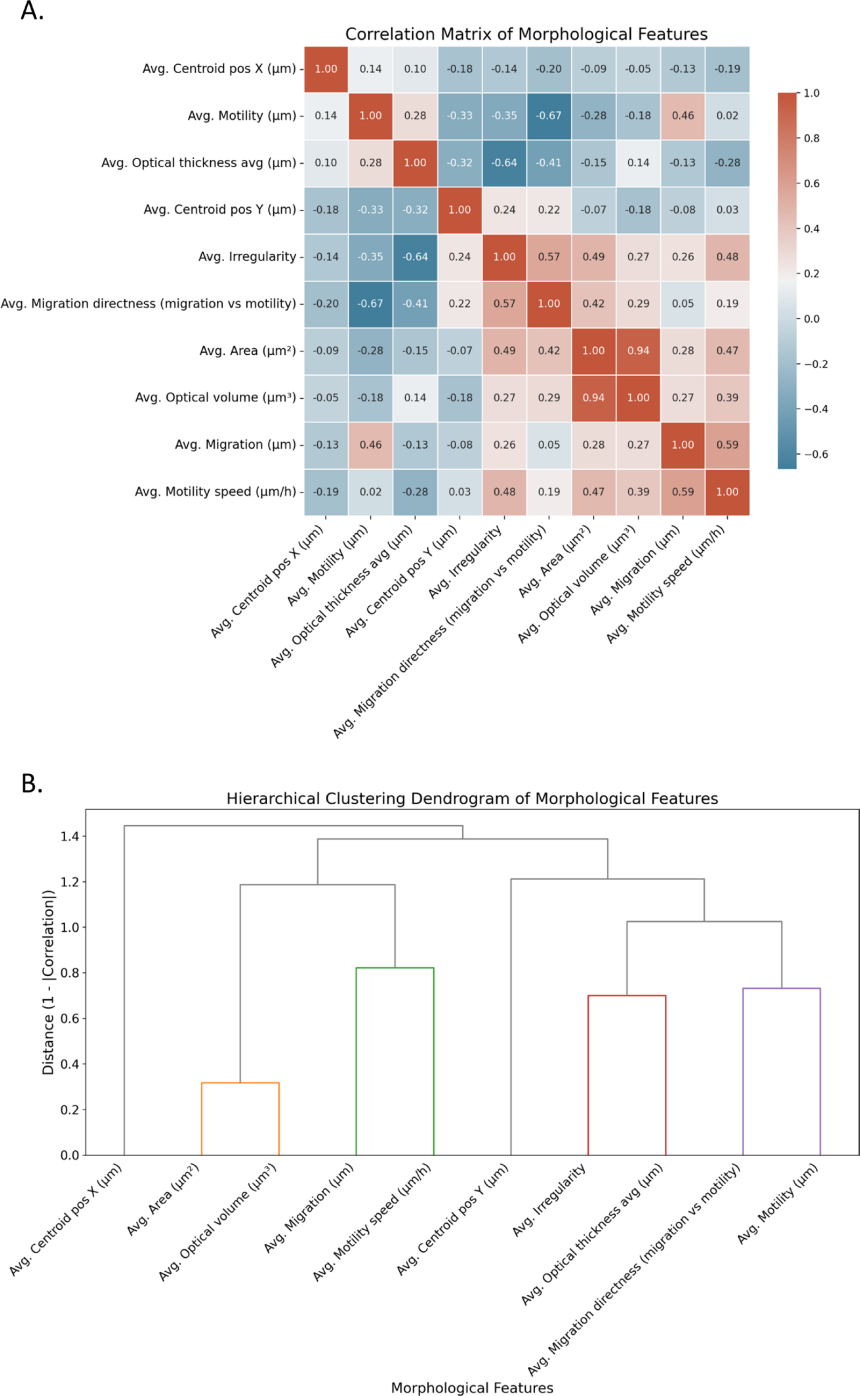
Analysis of morphological feature relationships. (A) Heatmap of correlation matrix. The heatmap presents the correlations between different features, with color intensity and hue representing the strength and direction of the correlation. Positive correlations are represented in red, and negative correlations are represented in blue. (B) Hierarchical clustering dendrogram of morphological features. The dendrogram illustrates the relationships between features based on similarity, with features that are more closely related positioned nearer to each other on the branching structure.

A Pearson correlation heatmap generated to visualize relationships among key morphological and functional features across both MCF10A and MDA‐MB‐231 cell lines captures overall trends by combining data from both cell lines rather than computing correlations separately, Figure 5A. The parameters that were assessed include average cellular area, optical volume, irregularity, motility speed, optical thickness, and migration characteristics. Our results reveal a strong positive correlation between average area and optical volume (*r* = 0.94), indicating that larger cells generally exhibit greater optical volume. This suggests that as cells increase in size, their internal complexity or functional properties may also expand. On the other hand, irregularity was moderately correlated with migration directness (*r* = 0.57), suggesting that cells with more irregular morphologies tend to exhibit more directed migration. However, this trend may be context‐dependent, as the strength of these relationships likely varies between cell lines. Motility speed exhibited only weak correlations with most features, with its highest correlation observed with average irregularity (*r* = 0.59). This indicates that while cells with higher motility speed may exhibit slight increases in irregularity ability, the relationship is not particularly strong. A moderate positive correlation (*r* = 0.19) was observed between motility and migration directness, suggesting that increased motility is somewhat associated with a more directed migration path, though additional factors may contribute to this relationship. These findings provide valuable insights into the structural and migratory properties of these two cell lines, emphasizing the interplay between morphology and function in cellular behavior. The dendrogram reveals distinct clustering patterns among cellular morphological features, with cell area and optical volume forming the most closely related pair (*r* = 0.94). Cell irregularity and optical thickness exhibit a strong negative correlation (*r* = −0.64), while migration directness and motility distance cluster separately from migration and motility speed, indicating distinct aspects of cellular movement. Centroid positions form independent clusters with minimal correlation to other morphological parameters, suggesting that spatial positioning operates independently from other cellular characteristics, Figure 5B.

### 3.6 Multiparametric Analysis Reveals Distinct Phenotypic Signatures Between Normal and Metastatic Breast Cell Lines

**FIGURE 6:**
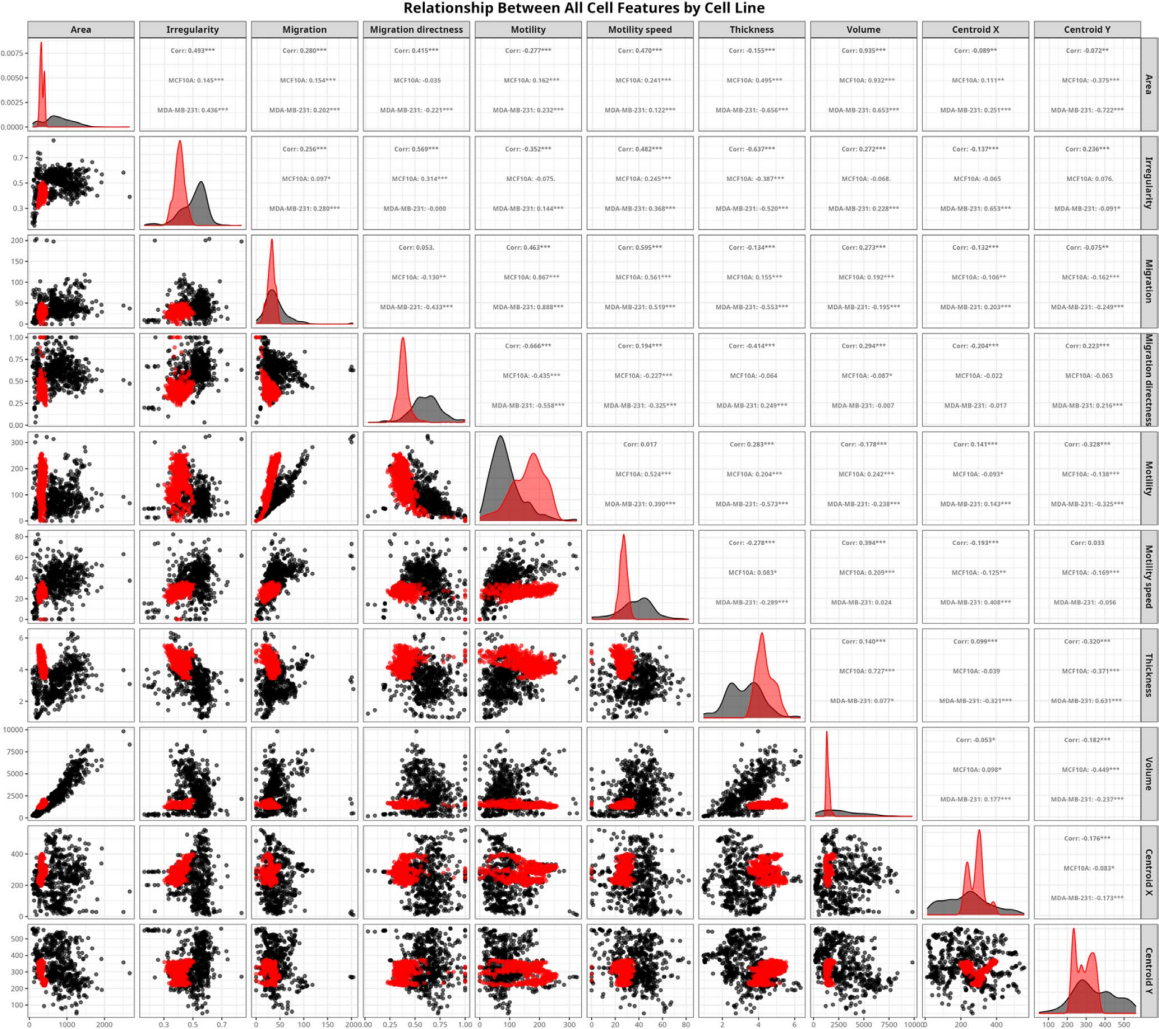
Scatter plot matrix illustrating pairwise relationships among cellular features for MCF10A (black) and MDA‐MB‐231 (red) cells: Average area (μm^2^), average irregularity, average migration (μm), average migration directness, average motility (μm), average motility speed (μm/h), average optical thickness (μm), average optical volume (μm^3^), and centroid *X* and *Y* positions. The diagonal panels show the distribution of each feature for both cell lines. Off‐diagonal panels display scatter plots for every possible feature pair, overlaid with a smoothed trend line. In the upper right corner of each scatter panel, three values are provided: The overall Pearson correlation coefficient (*r*) for all cells, the cell‐line–specific *r* values for MCF10A and MDA‐MB‐231, and the statistical significance of the overall correlation (*p < 0.05; **p < 0.01; ***p < 0.001).

Our comprehensive analysis of Pearson correlation coefficients between morphological and behavioral parameters revealed striking differences in the phenotypic organization of normal breast epithelial MCF10A and metastatic breast cancer MDA‐MB‐231 cells, Figure 6. These differences in correlation patterns provide quantitative evidence of the fundamental rewiring of cellular programs during metastatic transformation.

The most dramatic difference was observed in the relationship between cell area and optical thickness, which exhibited a complete reversal from a moderate positive correlation in MCF10A cells (*r* = 0.50) to a strong negative correlation in MDA‐MB‐231 cells (*r* = −0.66), representing a substantial difference (Δ*r* = −1.15). This finding indicates that while larger normal MCF10A cells tend to be thicker, MDA‐MB‐231 cells display an inverse relationship where increased thickness is associated with decreased spreading area—potentially reflecting a fundamental reorganization of cellular architecture in the metastatic phenotype.

Similarly, optical thickness showed reversed correlation patterns with multiple parameters in metastatic cells. The correlation between motility and optical thickness shifted from positive in MCF10A cells (*r* = 0.20) to strongly negative in MDA‐MB‐231 cells (*r* = −0.57; Δ*r* = −0.78), and migration similarly changed from a positive correlation with optical thickness (*r* = 0.16) to a negative one (*r* = −0.55; Δ*r* = −0.71). These reversals suggest that the three‐dimensional organization of metastatic cells fundamentally alters their motility behavior compared to normal epithelial cells.

Interestingly, there was a substantial weakening of the correlation between optical thickness and optical volume in MDA‐MB‐231 cells (*r* = 0.08) compared to MCF10A cells (*r* = 0.73; Δ*r* = −0.65). This decoupling of typically related parameters suggests that metastatic cells have developed a more complex relationship between their three‐dimensional structure and volume, potentially reflecting altered cytoskeletal organization or nuclear‐cytoplasmic ratios. The relationship between motility and optical volume also showed a notable shift from a positive correlation in MCF10A cells (*r* = 0.24) to a negative correlation in MDA‐MB‐231 cells (*r* = −0.24; Δ*r* = −0.48), further supporting the conclusion that metastatic transformation involves a comprehensive reorganization of how cellular structure relates to migratory behavior. Migration directness—a measure of the efficiency of cell movement—showed altered correlation patterns with several parameters. The correlation between irregularity and migration directness decreased from a moderate positive value in MCF10A cells (*r* = 0.31) to no correlation in MDA‐MB‐231 cells (*r* = 0.00; Δ*r* = −0.31), while the relationship between migration directness and optical thickness reversed from slightly negative (*r* = −0.06) to moderately positive (*r* = 0.25; Δ*r* = 0.31). Moreover, the negative correlation between migration and migration directness was substantially stronger in MDA‐MB‐231 cells (*r* = −0.43) compared to MCF10A cells (*r* = −0.13; Δ*r* = −0.30), indicating a more pronounced trade‐off between overall movement and directional persistence in metastatic cells. This may reflect enhanced exploratory behavior during invasion processes, where cells sacrifice directional efficiency for increased sampling of the microenvironment. Despite these substantial differences, certain core relationships remained relatively preserved between the cell lines. The strong positive correlation between migration and motility was maintained in both MCF10A (*r* = 0.87) and MDA‐MB‐231 cells (*r* = 0.89; Δ*r* = 0.02), suggesting that the fundamental coupling between these aspects of cell movement remains intact despite metastatic transformation. Similarly, the correlation between migration and motility speed showed only minor changes between MCF10A (*r* = 0.56) and MDA‐MB‐231 cells (*r* = 0.52; Δ*r* = −0.04), indicating that certain basic relationships in cellular motility machinery are conserved even as other aspects of the phenotype undergo substantial rewiring.

In general, our correlation analysis reveals that metastatic transformation involves not only changes in individual cellular parameters but also a comprehensive reorganization of the relationships between these parameters. The most dramatic differences involve optical thickness correlations, suggesting that the three‐dimensional organization of metastatic cells plays a central role in their altered phenotype.

### 3.7 Phenotypic Differences Between MCF10A and MDA‐MB‐231 Cell Lines

**FIGURE 7:**
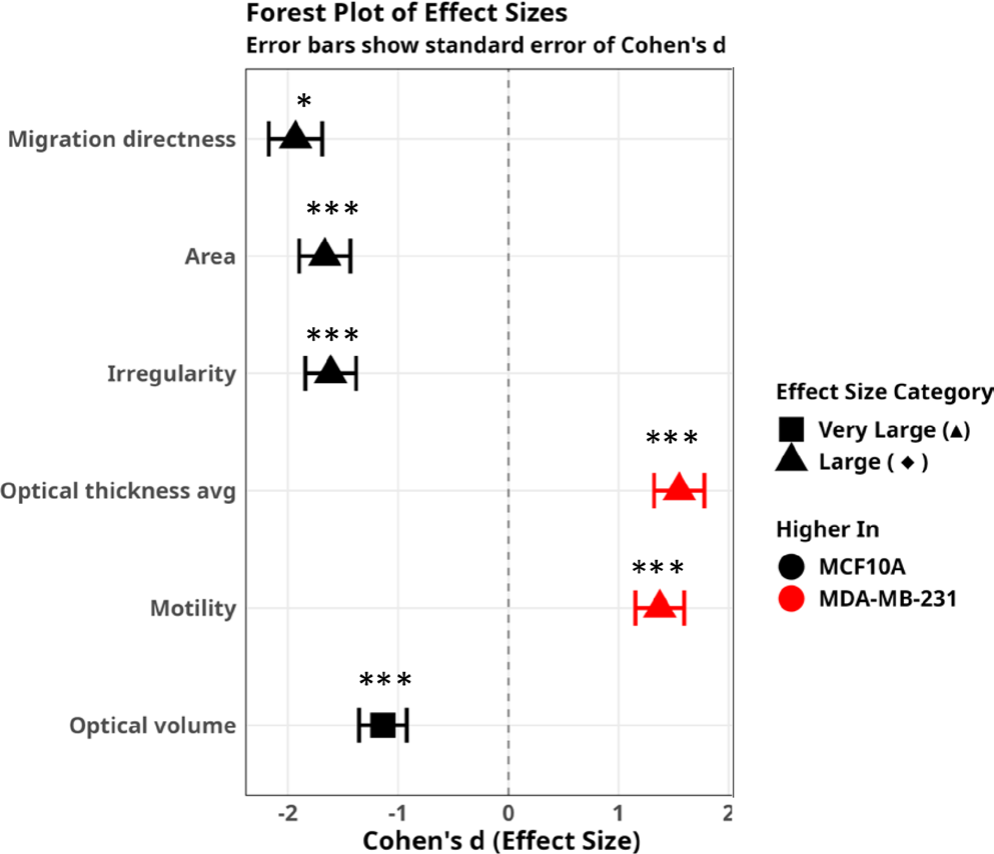
Forest plot of Cohen's *d* effect sizes for six key features. Each point represents the effect size (Cohen's *d*) comparing MCF10A and MDA‐MB‐231 cells; error bars denote ±1 SE, and asterisks indicate statistical significance (*p < 0.1;***p < 0.001). Black squares (■) indicate features higher in MCF10A; red circles (●) indicate features higher in MDA‐MB‐231.

A more comprehensive quantitative analysis of the phenotypic differences between the two cell lines was conducted using Cohen's d effect size measurements (Figure 7). We identified several key distinguishing features between these cell lines. Our analysis revealed that MCF10A cells display significantly higher migration directness (*d* = −1.93 ± 0.24), indicating more coordinated and directional movement patterns compared to MDA‐MB‐231 cells. Additionally, MCF10A cells exhibited greater area (*d* = −1.67 ± 0.23) and irregularity (*d* = −1.61 ± 0.23), reflecting their characteristic morphological structure. These cells also maintained larger optical volume (*d* = −1.14 ± 0.22), suggesting differences in three‐dimensional cellular organization between the two cell types. Conversely, MDA‐MB‐231 cells demonstrated markedly increased optical thickness (*d* = 1.55 ± 0.23) and motility (*d* = 1.37 ± 0.22). All observed differences were classified as very large (|*d*| ≥ 1.2) or large (0.8 ≤ |*d*| < 1.2) according to established effect size conventions, highlighting the robust and biologically significant phenotypic distinctions between these cell lines. These findings provide quantitative support for the cellular transformation that occurs during cancer progression, where cells transition from a structured, directional phenotype to a more amorphous, invasive state. The clear separation of phenotypic features between MCF10A and MDA‐MB‐231 cells demonstrates the utility of our multiparametric approach in characterizing cellular states relevant to cancer progression. These quantitative metrics may serve as valuable biomarkers for assessing metastatic potential in future studies of breast cancer cell biology and therapeutic interventions.

## 4 Discussion

The comprehensive analysis of the MCF10A and MDA‐MB‐231 cell lines presented in this study elucidates fundamental distinctions in morphology and dynamic cellular behaviors, which are critical for understanding the mechanisms underlying non‐tumorigenic and aggressive cancer phenotypes. The multidimensional scaling, PCA, and t‐SNE analyses (Figures 1B and 2A) underscore the distinct clustering and variability between the MCF10A and MDA‐MB‐231 cell lines. The PCA results indicate some overlap in cellular features, suggesting shared biological processes or cellular functions. However, the clearer separation observed in the t‐SNE plots highlights profound differences likely due to unique adaptive mechanisms inherent to the aggressive MDA‐MB‐231 cells [26, 27, 28]. These findings align with previous research indicating that triple‐negative breast cancer cells often exhibit distinct metastatic capabilities and molecular profiles compared to non‐tumorigenic cells [29, 30, 31]. This study sheds light on potential biomarkers associated with TNBC aggressiveness. Specifically, features such as enhanced motility and increased optical thickness in MDA‐MB‐231 cells consistently differentiate them from non‐tumorigenic MCF10A cells. These phenotypic adaptations, which are critical for tissue invasion and metastasis, highlight motility and optical properties as promising biomarkers for identifying aggressive TNBC cells.

Our comparative analysis of cell dynamics (Figure 3) demonstrated that despite their larger size, MCF10A cells exhibit less motility than MDA‐MB‐231 cells, a characteristic potentially advantageous in stable tissue environments but a disadvantage in metastatic potential. The greater cellular area and lower optical thickness of MCF10A cells suggest a higher degree of cellular complexity, which has been associated with enhanced metabolic and homeostatic functions [32, 33].

Conversely, the smaller and more motile MDA‐MB‐231 cells may exploit these traits to effectively navigate and invade new tissue microenvironments, as suggested by their higher motility and lower migration directness scores. The statistical analysis further reinforces these findings by quantitatively establishing the link between motility, size, and invasive potential. Independent *t*‐tests revealed highly significant differences across all measured metrics, with *p*‐values below 10^−18^, confirming strong statistical separation between the MCF10A and MDA‐MB‐231 cell lines. The large effect size for Avg_Directness in particular supports the hypothesis that the invasive potential of MDA‐MB‐231 cells is associated with their less directed, exploratory migration patterns, a hallmark of metastatic behavior [34]. While this study focuses on the MCF10A and MDA‐MB‐231 cell lines, which are well‐established models for non‐tumorigenic and TNBC research, we acknowledge that TNBC is a highly heterogeneous disease with multiple molecular subtypes [35]. As such, our findings should be interpreted within this context. The correlation between smaller cell size, increased motility, and invasive potential observed in MDA‐MB‐231 cells is consistent with prior studies across multiple TNBC models [36]. Similar patterns have been reported in other aggressive TNBC cell lines, suggesting that these traits may be generalizable markers of metastatic potential. However, future studies should validate these observations across a broader panel of TNBC cell lines, including those representing different molecular subtypes. Our findings suggest that quantitative motility‐based metrics, combined with real‐time imaging, could provide functional insights into TNBC cell behavior, in particular cellular metastasis [37]. Future investigations using patient‐derived organoids or clinical samples could further assess the applicability of these findings in predicting TNBC progression and metastatic potential. These quantitative findings align with previous research linking smaller cell size and increased motility to higher invasiveness in aggressive cancer phenotypes [37, 38].

Moreover, the observed increase in motility in MDA‐MB‐231 cells, could be driven by cytoskeletal reorganization and motility machinery, representing a mechanism that could be disrupted to limit metastatic dissemination [39]. Similarly, the increased optical thickness in these cells, indicative of structural adaptations, could inform strategies to target intracellular components crucial for TNBC survival and invasion. Such an increase in optical thickness may indicate structural variations, potentially linked to differences in intracellular composition, cytoskeletal organization, or metabolic activity. While optical thickness is a label‐free measurement that reflects the phase shift of light passing through cells, its biological implications remain an area of active investigation. The central reduction in optical thickness corresponds to the lower refractive index of the nucleus, as measured by DHM (nuclear RI ≈ 1.34 vs. cytoplasmic RI ≈ 1.38–1.39 [40, 41]). In contrast, perinuclear and peripheral cytoplasmic regions—enriched in mitochondria, endoplasmic reticulum, and actin networks—exhibit higher phase shifts due to their greater refractive index [42, 43], accounting for the brighter rims seen in Figure 1. Recent findings suggest that dynamic fluctuations in optical thickness are strongly correlated with intracellular biological processes that drive motility and invasion through extracellular matrices [44]. This indicates that higher optical thickness in MDA‐MB‐231 cells may contribute to their mechanical adaptability, perhaps allowing them to better navigate and invade complex microenvironments. Furthermore, the ability of optical thickness measurements to provide real‐time insights into invasiveness may present a useful alternative or complement to traditional single‐cell motility tracking methods. However, further research is needed to fully establish how optical thickness mechanistically contributes to TNBC progression at the molecular level. Such interventions could inhibit the ability of TNBC cells to adapt to and invade diverse microenvironments, offering novel approaches to mitigating TNBC progression.

The temporal dynamics explored in Figure 4 reveal that the aggressive characteristics of the MDA‐MB‐231 cells are consistently expressed over time, with significant differences in motility and migration patterns compared to MCF10A cells. These results support the hypothesis that intrinsic cellular mechanisms, such as cytoskeletal reorganization and motility machinery, are fundamentally different in these cell lines. The consistency of these patterns over time underscores the stable phenotypic adaptations that likely contribute to the invasive and metastatic nature of MDA‐MB‐231 cells [45, 46].

Our correlative analysis (Figures 5, 6, 7) provides further insights into how morphological and functional parameters interrelate within and between these cell lines. The positive correlations between cellular area and optical volume within both cell lines reinforce that cell size and internal complexity are closely linked, affecting cellular functions such as migration and metabolic activity [47, 48]. However, the differential correlation patterns in migration directness and motility between MCF10A and MDA‐MB‐231 cells suggest distinct navigation strategies, Figure 6. While MCF10A cells maintain more structured movement patterns, MDA‐MB‐231 cells exhibit a trade‐off between motility and directional persistence, likely reflecting an adaptive strategy for tissue invasion in metastatic cells.

In order to distinguish non‐tumorigenic and TNBC cell lines, several established methods are widely used, including flow cytometry, fluorescence microscopy, and molecular profiling techniques such as gene expression and proteomics [49]. While these approaches are effective in identifying specific cellular markers or molecular pathways, they often require fluorescent labeling, genetic modification, or extensive sample preparation, which can perturb natural cell behavior or limit the ability to monitor real‐time dynamics. In contrast, our approach employs digital holographic microscopy (DHM) in combination with advanced analytical techniques like PCA and t‐SNE, offering unique advantages. DHM is a label‐free imaging technique that preserves the integrity of live cells, allowing for longitudinal observations of cellular behaviors such as motility and morphological changes under near‐physiological conditions [15, 20, 50, 51]. This combination not only provides a deeper understanding of dynamic cellular processes but also facilitates the identification of novel biomarkers and therapeutic targets with minimal disruption to cell behavior. These differences were not only visually apparent in the PCA and t‐SNE plots but were also statistically validated through independent *t*‐tests, confirming that the differences at the cellular level between the two cell lines are highly significant. This statistical validation ensures that the observed trends are not random but are reflective of intrinsic biological characteristics of these cells.

Furthermore, our findings contribute to elucidating cellular homeostasis by demonstrating how distinct morphological and functional features of MCF10A and MDA‐MB‐231 cells relate to their ability to maintain equilibrium. The higher migration directness and larger cellular area observed in MCF10A cells suggest a homeostatic regulatory mechanism that supports tissue integrity. Their structured migration pattern likely facilitates epithelial maintenance, while their larger cell size may reflect enhanced cytoskeletal stability, properties associated with normal breast epithelial function [52, 53]. Future studies should explore the molecular underpinnings of these behaviors, including the role of mechanotransduction pathways and metabolic adaptation in maintaining cellular equilibrium. Importantly, while this study provides detailed insights into TNBC aggressiveness by comparing non‐tumorigenic MCF10A with the highly metastatic MDA‐MB‐231 cell line, TNBC is a heterogeneous disease composed of multiple subtypes. The use of only one non‐tumorigenic and one tumorigenic cell line limits the extent to which these findings can be generalized across all TNBC subtypes. Therefore, future work should expand on these findings by incorporating additional TNBC cell lines to evaluate whether these distinct phenotypic characteristics persist across a broader range of breast cancer models. This study provides statistically validated insights into TNBC cellular behaviors, highlighting differences in size, migration, and motility between MCF10A and MDA‐MB‐231 cells. However, given TNBC's heterogeneity, future work incorporating additional cell lines is essential to determine whether these phenotypic differences are representative of all TNBC subtypes. By refining our understanding of these behaviors, we can better characterize the factors that contribute to TNBC aggressiveness and identify potential targets for therapeutic intervention.

In conclusion, our study reveals critical insights into the differential phenotypic and dynamic behaviors of non‐tumorigenic and triple‐negative breast cancer cell lines. These differences are not only pivotal for understanding cancer biology but also for developing targeted therapeutic strategies. Features such as enhanced motility, reduced migration directness, and increased optical thickness in TNBC cells serve as potential biomarkers for aggressiveness. Additionally, therapeutic interventions aimed at disrupting motility machinery or targeting intracellular structural adaptations could provide novel avenues to mitigate TNBC progression. Future research should focus on unraveling the molecular drivers behind these phenotypic behaviors and exploring how these insights could translate into clinical interventions. Moreover, given the increasing role of AI in cancer research, integrating machine learning models to analyze high‐dimensional cellular imaging data could further enhance the predictive accuracy of cancer cell characterization. AI‐driven techniques, such as deep learning‐based feature extraction and automated classification, could be employed to refine the detection of subtle morphological patterns that define TNBC aggressiveness. This integration of AI could facilitate real‐time analysis of cellular behaviors, improving diagnostic precision and aiding in the development of personalized treatment strategies. While this study identifies key phenotypic differences between non‐tumorigenic and TNBC cells, additional studies using a more diverse panel of TNBC cell lines are needed to confirm the broader applicability of these findings. These results lay an important foundation for future investigations into TNBC behavior and therapeutic targeting strategies.

## Author Contributions

Study conception and design: B.X. Acquisition of data: B.X., Y.A.A., A.J.M. Analysis and interpretation of data: B.X., Y.A.A., E.R.S., A.J.M. Drafting of the manuscript: B.X. The Authors read and approved the final manuscript.

## Ethics Statement

Ethical approval was not required for this study, as all experiments were conducted using established cell lines purchased from a commercial vendor.

## Conflicts of Interest

The authors declare no conflicts of interest.

## Supporting information


**Figure S1.** Digital holographic microscopy (DHM) images of (A) MCF10A and (B) MDA‐MB‐231 cells with computationally segmented boundaries overlaid. Cell borders were extracted from raw phase‐shift maps using gradient‐based edge detection to enhance interpretability while preserving the label‐free nature of DHM imaging. This figure complements Figure 1 by providing clearer visualization of individual cell outlines.

**Table S1.** Optical thickness and density metrics for MCF10A and MDA‐MB‐231 cells.

## Data Availability

All data supporting the findings of this study are available from the corresponding author upon reasonable request.
