# Supplementary figures and images for: Dissecting Morphological and Functional Dynamics of Non‐Tumorigenic and Triple‐Negative Breast Cancer Cell Lines Using PCA and t‐SNE Analysis
Source: Cancer Rep (Hoboken). 2025 Jun 27;8(7):e70257. doi: 10.1002/cnr2.70257 (PMC12203836; doi:10.1002/cnr2.70257)

A. MCF10A

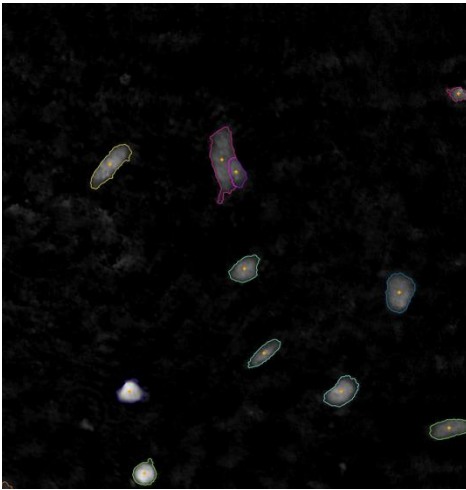

B. MDA-MB-231

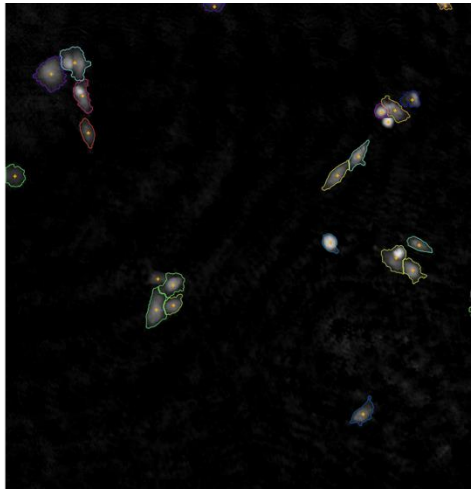

Figure S1

Supplement: Supplementary file 1 — Figure S1. Digital holographic microscopy (DHM) images of (A) MCF10A and (B) MDA‐MB‐231 cells with computationally segmented boundaries overlaid. Cell borders were extracted from raw phase‐shift maps using gradient‐based edge detection to enhance interpretability while preserving the label‐free nature of DHM imaging. This figure complements Figure 1 by providing clearer visualization of individual cell outlines. [file CNR2-8-e70257-s002.pdf]
